# Impact of *PSCA* Variation on Gastric Ulcer Susceptibility

**DOI:** 10.1371/journal.pone.0063698

**Published:** 2013-05-21

**Authors:** Chizu Tanikawa, Keitaro Matsuo, Michiaki Kubo, Atsushi Takahashi, Hidemi Ito, Hideo Tanaka, Yasushi Yatabe, Kenji Yamao, Naoyuki Kamatani, Kazuo Tajima, Yusuke Nakamura, Koichi Matsuda

**Affiliations:** 1 Laboratory of Molecular Medicine, Human Genome Center, Institute of Medical Science, The University of Tokyo, Tokyo, Japan; 2 Division of Epidemiology and Prevention, Aichi Cancer Center Research Institute, Aichi, Japan; 3 Department of Pathology and Molecular Diagnostics, Aichi Cancer Center Research Institute, Aichi, Japan; 4 Department of Gastroenterology, Aichi Cancer Center Research Institute, Aichi, Japan; 5 Center for Genomic Medicine, The Institute of Physical and Chemical Research (RIKEN), Kanagawa, Japan; 6 Departments of Medicine and Surgery, and Center for Personalized Therapeutics, The University of Chicago, Chicago, Illinois, United States of America; Tor Vergata University of Rome, Italy

## Abstract

Peptic ulcer is one of the most common gastrointestinal disorders with complex etiology. Recently we conducted the genome wide association study for duodenal ulcer and identified disease susceptibility variations at two genetic loci corresponding to the *Prostate stem cell antigen* (*PSCA*) gene and the *ABO blood group* (*ABO*) gene. Here we investigated the association of these variations with gastric ulcer in two Japanese case-control sample sets, a total of 4,291 gastric ulcer cases and 22,665 controls. As a result, a C-allele of rs2294008 at *PSCA* increased the risk of gastric ulcer with odds ratio (OR) of 1.13 (*P* value of 5.85×10^−7^) in an additive model. On the other hand, SNP rs505922 on *ABO* exhibited inconsistent result between two cohorts. Our finding implies presence of the common genetic variant in the pathogenesis of gastric and duodenal ulcers.

## Introduction

Peptic ulcer is the most common disease in the gastrointestinal tract with symptoms of nausea, vomiting, and abdominal pain, and sometimes causes bleeding and perforation with acute peritonitis. Lifetime prevalence of peptic ulcer is 10–15% in the Japanese and 4–10% in Caucasians [1]–[3]. Approximately 70% of gastric ulcer patients and 90% of duodenal ulcer patients are associated with *H. pylori* infection [4]. Since eradication of *H. pylori* by antibiotics in combination with proton pomp inhibitor can effectively cure peptic ulcer [5], *H. pylori* is shown to be the major cause of peptic ulcer. Although nearly 50% of individuals on the earth are infected with *H. pylori,* most of them remain asymptomatic indicating that the clinical outcome after the *H. pylori* infection varies substantially between individuals. These inter-individual diversities are affected by various factors including bacteria subtypes, host response, and their interaction. *Duodenal ulcer promoting gene A* (*dupA*) in *H. pylori* was indicated to induce interleukin (IL)-8 that increases the risk of duodenal ulcer and decreases the risk of gastric cancer [6], [7]. Nonsteroidal anti-inflammatory drugs (NSAIDs) and smoking are known risk factors for peptic ulcer [8], [9]. In addition to these bacterial and environmental factors, host genetic factors had been implicated to have some roles in the risk of peptic ulcer. Proband-wise concordance rate of peptic ulcer in monozygotic twins was as high as 23.6% while that in dizygotic twins was 14.8%. Several candidate gene approaches revealed the possible association of genetic variations in *IL-6*, *IL-8*, *IL-10* [10], *TNF*, *LTA* [11], and *COX1* [12] with peptic ulcer risk.

In our previous genome wide association study (GWAS) of duodenal ulcer using a total of 7,035 cases and 25,323 controls, we identified the significant association of genetic variations at *PSCA* (*prostate stem cell antigen*) and the *ABO* blood group with duodenal ulcer [13]. The C allele of rs2294008 at *PSCA* increased the risk of duodenal ulcer (odds ratio (OR) of 1.84 with *P* value of 3.92×10^−33^) in a recessive model, while it decreased the risk of gastric cancer (OR of 0.79 with *P* value of 6.79×10^−12^) as reported previously [14]. Our functional analyses revealed that the T allele of SNP rs2294008 creates an upstream translational initiation codon and add the signal peptide sequences at the N-terminal portion, resulting in alteration of the protein subcellular localization from cytoplasm to cell surface. SNP rs505922 on *ABO* was also associated with duodenal ulcer in a recessive model (OR of 1.32 with *P* value of 1.15×10^−10^). Since *H. pylori* infection and non-steroidal anti-inflammatory drugs induce gastroduodenal mucosal injury which would cause duodenal and gastric ulcer, we examined the role of variants in the *PSCA* and *ABO* genes on gastric ulcer risk among Japanese population.

## Results

A total of 4,291 gastric ulcer cases and 22,665 controls without having the past history of duodenal ulcer or continuous NSAID intake were recruited from the BioBank Japan and the Aichi Cancer Center (**Table 1**). We then genotyped SNP rs2294008 and rs505922 in two case-control sample sets and examined the association with gastric ulcer in three genetic models (additive, recessive, and dominant model) (**Table 2**). To increase the statistical power of this study, we used subjects with either of 22 diseases as control samples. Therefore we evaluated the confounding effect of disease mix control samples used in this analysis. SNPs rs2294008 and rs505922 did not show significant association between case-mix controls (n = 19,884) and healthy volunteers (n = 2,781) (**Table S1**). In addition, both SNPs did not show the significant deviation from HWE (Hardy-Weinberg equilibrium) in each disease group. Therefore disease mix controls seem not to largely affect the association result in our analysis.

**Table 1:** Characteristics of study population.

Samples	Source	Platform	Number of samples	Female (%)		Age (mean +/– SD)
Gastric ulcer^a^	BioBank Japan	Illumina HumanHap 610	1,862	32.0	(%)	66.0+/–10.7
		Invader assay	2,004	35.3	(%)	66.5+/–11.7
	Aichi Cancer Center	TaqMan	425	48.7	(%)	55.6+/–12.3
Control^a^	BioBank Japan^b^	Illumina HumanHap 610	17,482	54.1	(%)	62.2+/–13.0
	BioBank Japan^b^	Illumina HumanHap 550	3,309	66.0	(%)	43.8+/–16.2
	Aichi Cancer Center	TaqMan	1,874	38.7	(%)	53.7+/–14.6

a Subjects with a history of gastric cancer or duodenal ulcer were excluded from cases and controls.

b Control samples consist of patients with colon cancer, breast cancer, diabetes, arteriosclerosis obliterans, atrial fibrillation, brain infarction, drug response, amyotrophic lateral sclerosis, liver cancer, liver cirrhosis, osteoporosis, fibroid, cervical cancer, chronic hepatitis B, ovarian cancer, pulmonary tuberculosis, keloid, drug eruption, hematological cancer, uterus cancer, heat cramp, endometriosis, and 907 healthy volunteers.

**Table 2:** Association of PSCA and ABO SNPs with gastric ulcer.

SNP		Case				Control				Additive				Recessive				Dominant			
Chr		CC	CT	TT	RAF^a^	CC	CT	TT	RAF^a^	*P* b	OR b (95% C.I.)		*P*_Het_ c	*P* b	OR b (95% C.I.)		*P*_Het_ c	*P* b	OR b (95% C.I.)		*P*_Het_ c
rs2294008 8q24/*PSCA*	BBJ	##	###	###	####	###	9577	###	####	2.55×10^−6^	###	(1.07–1.18)		6.12×10^−4^	1.18	(1.07–1.30)		3.52×10^−5^	###	(1.08–1.25)	
	ACC	70	201	154	####	235	917	722	####	9.09×10^−2^	###	(0.98–1.33)	####	3.10×10^−2^	1.38	(1.03–1.84)	####	0.380	###	(0.89–1.37)	0.657
	meta^d^									5.85×10^−7^	###	(1.08–1.18)		8.94×10^−5^	1.20	(1.09–1.31)		2.60×10^−5^	###	(1.08–1.24)	
rs505922 9q34/*ABO*	BBJ	##	###	###	####	###	####	###	####	9.39×10^−4^	###	(1.03–1.14)	6.91×10^−2^	7.72×10^−4^	1.13	(1.05–1.22)		5.26×10^−2^	###	(1.00–1.19)	7.90×10^−2^
	ACC	97	201	127	####	379	925	570	####	0.407	###	(0.81–1.09)		0.829	0.97	(0.77–1.23)	####	0.232	###	(0.67–1.10)	
	meta^d^									3.88×10^−3^	###	(1.02–1.12)		1.75×10^−3^	1.12	(1.04–1.20)		0.141	###	(0.98–1.15)	

We analyzed 4,291 gastric ulcer cases and 22,665 controls.

a rs2294008 (C allele) and rs505922 (T allele).

b P values were obtained using chi-square test. To calculate odds ratios (OR), non risk alleles were considered as references.

c Heterogeneity across two stages was assessed by Cochran Q test.

d OR and P values were obtained using the Mantel-Haenszel fixed-effects model in the meta analysis.

The results of association analyses revealed that gastric ulcer patients had a higher frequency of C allele at rs2294008 than the control group in both sets (39.7% vs 36.9% and 40.1% vs 37.0%, respectively). A meta-analysis of the two studies showed the significant association of rs2294008 in an additive model with no evidence of heterogeneity (P = 5.85×10^−7^ with OR of 1.13), although the association was not statistically significant among Aichi Cancer Center cohort probably due to smaller sample size. Risk alleles (C allele at rs2294008) in the two sample sets were consistent between duodenal ulcer and gastric ulcer, indicating the role of *PSCA* variation as common genetic factors for peptic ulcer. However impact of this variation on gastric ulcer risk was not as strong as those on duodenal ulcer reported previously [13].

On the other hands, SNP rs505922 showed inconsistent results between two cohorts. A T allele of rs505922 increased gastric ulcer risk in all three genetic models in BioBank Japan cohort. However, gastric ulcer patients exhibited lower frequency (53.5%) of a T allele than the healthy controls (55.1%) in the Aichi Cancer Center cohort. Therefore, further association analysis is essential to determine the role of *ABO* variations on gastric ulcer susceptibility.

Since we have genotyping results of 1,862 gastric ulcer cases and 17,482 controls analyzed by Illumina Human Hap610-Quad genechip, we conducted whole genome screening using these sample set. Although 62 SNPs exhibited suggestive associations with *P* values of less than 1×10^−4^, no SNPs cleared genome wide significant threshold (**Table S2** and **Figure S1**). Thus, our sample set did not have sufficient statistical power to detect gastric ulcer susceptibility loci by GWAS.

We also investigated the association of previously reported genes with gastric ulcer (**Table 3**). We selected 32 SNPs at five gene loci that had been genotyped by Illumina Human Hap610-Quad genechip. As a result, two loci at *LTA* and *PTGS1* indicated suggestive association (P = 1.64×10^−3^ and 0.0376), although these associations were not statistically significant after Bonferroni’s correction (*P*<0.00156 = 0.05/32). Thus further analyses are necessary to elucidate the role of these variations on gastric ulcer.

**Table 3:** Association of variations on candidate genes with Gastric ulcer.

SNP	Gene	relative loc	Chr	Position	Gastric ulcer		
					a *P*	b OR (95% C.I.)	
rs3024505	*IL10*	1044	1	2.05E+08	0.817	###	(0.71–1.31)
rs3024498	*IL10*	0	1	2.05E+08	0.569	###	(0.55–3.01)
rs1554286	*IL10*	0	1	2.05E+08	0.911	###	(0.93–1.08)
rs3021094	*IL10*	0	1	2.05E+08	0.178	###	(0.98–1.12)
rs3024490	*IL10*	0	1	2.05E+08	0.902	###	(0.93–1.07)
rs2222202	*IL10*	0	1	2.05E+08	0.975	###	(0.74–1.33)
rs1800896	*IL10*	−1058	1	2.05E+08	0.766	###	(0.87–1.20)
rs2844484	*LTA*	−3869	6	31644203	4.41×10^−2^	###	(1.00–1.15)
rs2009658	*LTA*	−1849	6	31646223	0.453	###	(0.94–1.14)
rs2844482	*LTA*	−326	6	31647746	0.484	###	(0.94–1.13)
rs1800683	*LTA*	−22	6	31648050	1.64×10^−3^	###	(0.83–0.96)
rs2229094	*LTA*	0	6	31648535	0.163	###	(0.98–1.16)
rs2229092	*LTA*	0	6	31648736	0.295	###	(0.90–1.42)
rs1041981	*LTA*	0	6	31648763	1.75×10^−3^	###	(0.83–0.96)
rs3093662	*TNF*	0	6	31652168	0.220	###	(0.91–1.51)
rs3093668	*TNF*	383	6	31654474	0.335	###	(0.87–1.51)
rs833068	*VEGFA*	0	6	43850505	0.427	###	(0.96–1.10)
rs833069	*VEGFA*	0	6	43850557	0.401	###	(0.96–1.10)
rs3025010	*VEGFA*	0	6	43855555	0.976	###	(0.93–1.07)
rs3025033	*VEGFA*	0	6	43859053	0.282	###	(0.96–1.14)
rs3025035	*VEGFA*	0	6	43859337	0.841	###	(0.93–1.09)
rs6900017	*VEGFA*	4261	6	43866463	9.77×10^−2^	###	(0.99–1.15)
rs2069837	*IL6*	0	7	22734552	0.728	###	(0.93–1.12)
rs2066992	*IL6*	0	7	22734774	0.953	###	(0.93–1.09)
rs1554606	*IL6*	0	7	22735232	0.813	###	(0.61–1.86)
rs10242595	*IL6*	2611	7	22740756	0.799	###	(0.87–1.11)
rs1236913	*PTGS1*	0	9	1.24E+08	0.389	###	(0.74–1.12)
rs1213266	*PTGS1*	0	9	1.24E+08	0.263	###	(0.81–1.06)
rs4836885	*PTGS1*	0	9	1.24E+08	0.964	###	(0.82–1.23)
rs6478565	*PTGS1*	0	9	1.24E+08	0.318	###	(0.78–1.08)
rs10306163	*PTGS1*	0	9	1.24E+08	0.119	###	(0.80–1.03)
rs10306202	*PTGS1*	1540	9	1.24E+08	3.76×10^−2^	###	(0.75–0.99)

We analyzed 1,862 gastric ulcer cases and 17,482 controls in this analysis. Chr.,chromosome; Postion in the NCBI Build 36.3.

a P values were calculated by Cochran Armitage trend test.

b OR, odds ratio was calculated by considering the major allele as the reference.

## Discussion

The development of gastric ulcer is determined by the interplay between gastric acid secretion and mucosal resistance, however their underling pathogenesis has not been fully elucidated. Gastric mucus, a gelatinous material secreted by gastric mucous cells, serves as an unstirred layer through which the diffusion of acid and pepsin is reduced. We here found that variation in the *PSCA* gene was significantly associated with gastric ulcer. PSCA was initially identified as a tumor antigen that was highly expressed in prostate, bladder, and pancreatic cancer tissues [15], [16]. Since tumor cells treated with anti-PSCA antibody exhibited a growth suppressive effect [17], [18], cell surface-PSCA is considered to play an important role in cell proliferation. In contrast, down-regulation of PSCA in gastric and esophageal cancer tissues was also reported [19], [20]. Thus the role of PSCA in carcinogenesis is still controversial [21]. These diverse effects of PSCA among various cancer types might be partially explained by the effect of genetic variation. Individuals carrying the T allele at rs2294008 express PSCA proteins with an additional fragment of nine amino acids at the N-terminal portion [13]. On the other hand, individuals carrying the C allele at rs2294008 express a shorter PSCA protein which lacks the signal peptide and is predicted to be localized in the cytoplasm without glycosylation [22]. We also found that the cytosolic shorter PSCA protein was more susceptible to proteasomal degradation than the long PSCA protein at the cell-surface. Since PSCA-derived peptides were reported to be a target of T-cell-based immunotherapy for advanced prostate cancer [23], the shorter PSCA protein would cause the activation of CD4-positive and/or CD8-positive T cells and subsequently promote epithelial mucosal injury [24]. In contrast, the long PSCA protein at the cell surface might facilitate mucosal repair by enhancing epithelial cell proliferation. In addition, T allele of SNP rs2294008 was shown to be associated with higher mRNA and protein expression [25]. Thus the impact of PSCA on gastric ulcer and carcinogenesis could be regulated by the *PSCA* variation.

*H. pylori* plays an important role in the development of gastritis, peptic ulcers, and gastric cancer, and the eradication of *H. pylori* was shown to reduce the recurrence of gastric ulcer [26] and prevent the onset of gastric cancer [27]. Since vertical transmission during childhood is the major source of infection, family history of *H. pylori* infection or *H. pylori*-related diseases is a risk factor for *H. pylori* infection [28]–[30]. In addition, recent accumulated evidences revealed a number of risk factors of gastric cancer (T allele at rs2294008, blood type A, decreased gastric acid, intake of proton pump inhibitor/H_2_ blocker, and *CagA* in *H. pylori* [31]) or peptic ulcer (C allele at rs2294008, blood type O, NSAID intake, *dupA* in *H. pylori*) [32]. In addition, *CYP2C19* genotype was associated with the response to triple anti-*H. pylori* therapy including proton pump inhibitor [33]. However, our previous analysis revealed that SNP rs2294008 and rs505922 did not associated with *H. pylori* prevalence [13]. Taking the above information into account, the estimation of disease risk and drug efficacy would enable us to determine the appropriate treatment protocol for *H. pylori* carriers.

Here we found that *PSCA* variant was significantly associated with gastric ulcer. In our previous analysis, PSCA variation did not associate with *H. pylori* prevalence [13]. Since *H. pylori* infection was associated with many diseases such as MALT lymphoma [34], idiopathic thrombocytopenic purpura [35], atrophic gastritis [36], and NSAID-induced gastric ulcer, it is very interesting to evaluate the effect of *PSCA* variation on these diseases. We hope our findings would contribute to the elucidation of disease pathogenesis as well as to the establishment of personalized medical treatments in the future.

## Methods

### Ethics Statement

This research project was approved by the ethical committees at the University of Tokyo, RIKEN, and Aichi Cancer Center. All participants provided written informed consent as approved by the ethical committees of the University of Tokyo and Aichi Cancer Center.

### Study participants

The demographic details of study participants are summarized in Table 1. A total of 3,866 gastric ulcer patients, and 20,791 gastric ulcer negative controls were obtained from BioBank Japan that was initiated in 2003 with the funding from the Ministry of Education, Culture, Sports, Science and Technology, Japan [37]. In the BioBank Japan Project, DNA and serum of patients with 47 diseases were collected through collaborating network of 66 hospitals throughout Japan. The list of participating hospitals is shown in the following website (http://biobankjp.org/plan/member_hospital.html). A total of 425 gastric ulcer cases and 1,874 healthy controls were obtained from the Aichi Cancer Center. The diagnosis of gastric ulcer was based on clinical, endoscopic, and histological features. List of disease-mix control samples used in this study was shown in **Table S1**. We excluded patients with duodenal ulcer or gastric cancer from both cases and controls. Deregulation of PSCA was reported in many types of malignancy such as prostate, pancreatic, lung, bladder, gastric, cholangiocarcinoma, and esophageal cancer [14]–[16], [20], [38], [39]. In addition, *ABO* locus was previously shown to be associated with various diseases such as myocardial infarction and pancreatic cancer [40], [41]. Therefore, we excluded subjects with these diseases from case mix controls. We also excluded the subjects with continuous NSAID intake.

### SNP Genotyping

Genotyping platforms used in this study are shown in Table 1. A total of 1,862 gastric ulcer cases and 20,791 gastric ulcer negative control samples were genotyped with Illumina Human Hap610-Quad or with Human Hap550v3. The other samples were genotyped by the Invader assay system (Third Wave Technologies, Madison, WI) or Taqman assay.

### Statistical Analysis

The association of SNPs rs2294008 and rs505922 with gastric ulcer was tested by chi-square test. The Odds ratios were calculated by considering the protective allele as the reference allele. The association of SNPs genotyped by Illumina Human Hap610-Quad with gastric ulcer was tested by multivariate logistic regression analysis upon adjusting for age at recruitment and gender using PLINK [42]. Heterogeneity across two stages was examined by Cochran Q test [43].

## Supporting Information

Figure S1 **Manhattan plot showing the genome-wide P values of association.** The P values were obtained by logistic regression analysis upon adjustment for age and gender. The y-axis represents the –log10 P values of 480,566 SNPs, and their chromosomal positions are shown on x-axis.
(TIF)

Table S1 **Genotype frequency of two SNPs in disease mix controls.**
(DOCX)

Table S2 **The result of association analysis of Gastric ulcer in GWAS.**
(DOCX)

## References

[1] Schlemper RJ, van der Werf SD, Vandenbroucke JP, Biemond I, Lamers CB (1993) Peptic ulcer, non-ulcer dyspepsia and irritable bowel syndrome in The Netherlands and Japan. Scand J Gastroenterol Suppl 200: 33–41. 10.3109/00365529309101573 8016569

[2] Araki S, Goto Y (1985) Peptic ulcer in male factory workers: a survey of prevalence, incidence, and aetiological factors. J Epidemiol Community Health 39: 82–85. 3989441 10.1136/jech.39.1.82 PMC1052408

[3] Schlesinger PK, Robinson B, Layden TJ (1992) Epidemiology considerations in peptic ulcer disease. J Assoc Acad Minor Phys 3: 70–77. 1498525

[4] Marshall BJ (1994) Helicobacter pylori. Am J Gastroenterol 89: S116–128. 8048402

[5] Hopkins RJ, Girardi LS, Turney EA (1996) Relationship between Helicobacter pylori eradication and reduced duodenal and gastric ulcer recurrence: a review. Gastroenterology 110: 1244–1252. 8613015 10.1053/gast.1996.v110.pm8613015

[6] Lu H, Hsu PI, Graham DY, Yamaoka Y (2005) Duodenal ulcer promoting gene of Helicobacter pylori. Gastroenterology 128: 833–848. 15825067 10.1053/j.gastro.2005.01.009 PMC3130061

[7] Argent RH, Burette A, Miendje Deyi VY, Atherton JC (2007) The presence of dupA in Helicobacter pylori is not significantly associated with duodenal ulceration in Belgium, South Africa, China, or North America. Clin Infect Dis 45: 1204–1206. 17918084 10.1086/522177

[8] Wolfe MM, Lichtenstein DR, Singh G (1999) Gastrointestinal toxicity of nonsteroidal antiinflammatory drugs. N Engl J Med 340: 1888–1899. 10369853 10.1056/NEJM199906173402407

[9] Chen TS, Lee YC, Li FY, Chang FY (2005) Smoking and hyperpepsinogenemia are associated with increased risk for duodenal ulcer in Helicobacter pylori-infected patients. J Clin Gastroenterol 39: 699–703. 16082280 10.1097/01.mcg.0000173854.55172.ee

[10] Kang JM, Kim N, Lee DH, Park JH, Lee MK, et al (2009) The effects of genetic polymorphisms of IL-6, IL-8, and IL-10 on Helicobacter pylori-induced gastroduodenal diseases in Korea. J Clin Gastroenterol 43: 420–428. 19077731 10.1097/MCG.0b013e318178d1d3

[11] Lanas A, García-González MA, Santolaria S, Crusius JB, Serrano MT, et al (2001) TNF and LTA gene polymorphisms reveal different risk in gastric and duodenal ulcer patients. Genes Immun 2: 415–421. 11781708 10.1038/sj.gene.6363798

[12] Arisawa T, Tahara T, Shibata T, Nagasaka M, Nakamura M, et al (2007) Association between genetic polymorphisms in the cyclooxygenase-1 gene promoter and peptic ulcers in Japan. Int J Mol Med 20: 373–378. 17671743

[13] Tanikawa C, Urabe Y, Matsuo K, Kubo M, Takahashi A, et al.. (2012) A genome-wide association study identifies two susceptibility loci for duodenal ulcer in the Japanese population. Nat Genet 44: 430–434, S431–432. 10.1038/ng.1109 22387998

[14] Sakamoto H, Yoshimura K, Saeki N, Katai H, Shimoda T, et al (2008) Genetic variation in PSCA is associated with susceptibility to diffuse-type gastric cancer. Nat Genet 40: 730–740. 18488030 10.1038/ng.152

[15] de Nooij-van Dalen AG, van Dongen GA, Smeets SJ, Nieuwenhuis EJ, Stigter-van Walsum M, et al (2003) Characterization of the human Ly-6 antigens, the newly annotated member Ly-6K included, as molecular markers for head-and-neck squamous cell carcinoma. Int J Cancer 103: 768–774. 12516096 10.1002/ijc.10903

[16] Gu Z, Thomas G, Yamashiro J, Shintaku IP, Dorey F, et al (2000) Prostate stem cell antigen (PSCA) expression increases with high gleason score, advanced stage and bone metastasis in prostate cancer. Oncogene 19: 1288–1296. 10713670 10.1038/sj.onc.1203426

[17] Marra E, Uva P, Viti V, Simonelli V, Dogliotti E, et al (2010) Growth delay of human bladder cancer cells by Prostate Stem Cell Antigen downregulation is associated with activation of immune signaling pathways. BMC Cancer 10: 129. 20374648 10.1186/1471-2407-10-129 PMC2858747

[18] Gu Z, Yamashiro J, Kono E, Reiter RE (2005) Anti-prostate stem cell antigen monoclonal antibody 1G8 induces cell death in vitro and inhibits tumor growth in vivo via a Fc-independent mechanism. Cancer Res 65: 9495–9500. 16230414 10.1158/0008-5472.CAN-05-2086

[19] Study Group of Millennium Genome Project for C, Sakamoto H, Yoshimura K, Saeki N, Katai H, et al (2008) Genetic variation in PSCA is associated with susceptibility to diffuse-type gastric cancer. Nat Genet 40: 730–740. 18488030 10.1038/ng.152

[20] Bahrenberg G, Brauers A, Joost HG, Jakse G (2000) Reduced expression of PSCA, a member of the LY-6 family of cell surface antigens, in bladder, esophagus, and stomach tumors. Biochem Biophys Res Commun 275: 783–788. 10973799 10.1006/bbrc.2000.3393

[21] Saeki N, Gu J, Yoshida T, Wu X (2010) Prostate stem cell antigen: a Jekyll and Hyde molecule? Clin Cancer Res 16: 3533–3538. 20501618 10.1158/1078-0432.CCR-09-3169 PMC2905486

[22] Nakai K, Kanehisa M (1992) A knowledge base for predicting protein localization sites in eukaryotic cells. Genomics 14: 897–911. 1478671 10.1016/S0888-7543(05)80111-9 PMC7134799

[23] Raff AB, Gray A, Kast WM (2009) Prostate stem cell antigen: a prospective therapeutic and diagnostic target. Cancer Lett 277: 126–132. 18838214 10.1016/j.canlet.2008.08.034 PMC2680000

[24] Ohara T, Morishita T, Suzuki H, Masaoka T, Ishii H (2003) Perforin and granzyme B of cytotoxic T lymphocyte mediate apoptosis irrespective of Helicobacter pylori infection: possible act as a trigger of peptic ulcer formation. Hepatogastroenterology 50: 1774–1779. 14696402

[25] Kohaar I, Porter-Gill P, Lenz P, Fu YP, Mumy A, et al (2013) Genetic variant as a selection marker for anti-prostate stem cell antigen immunotherapy of bladder cancer. J Natl Cancer Inst 105: 69–73. 23266392 10.1093/jnci/djs458 PMC3536639

[26] Marshall BJ, Goodwin CS, Warren JR, Murray R, Blincow ED, et al (1988) Prospective double-blind trial of duodenal ulcer relapse after eradication of Campylobacter pylori. Lancet 2: 1437–1442. 2904568 10.1016/s0140-6736(88)90929-4

[27] Fukase K, Kato M, Kikuchi S, Inoue K, Uemura N, et al (2008) Effect of eradication of Helicobacter pylori on incidence of metachronous gastric carcinoma after endoscopic resection of early gastric cancer: an open-label, randomised controlled trial. Lancet 372: 392–397. 18675689 10.1016/S0140-6736(08)61159-9

[28] Leung WK, Ng EK, Lam CC, Chan KF, Chan WY, et al (2006) Helicobacter pylori infection in 1st degree relatives of Chinese gastric cancer patients. Scand J Gastroenterol 41: 274–279. 16497613 10.1080/00365520510024269

[29] Brenner H, Bode G, Boeing H (2000) Helicobacter pylori infection among offspring of patients with stomach cancer. Gastroenterology 118: 31–35. 10611151 10.1016/s0016-5085(00)70411-2

[30] Brenner H, Rothenbacher D, Bode G, Adler G (1998) Parental history of gastric or duodenal ulcer and prevalence of Helicobacter pylori infection in preschool children: population based study. BMJ 316: 665. 9522790 10.1136/bmj.316.7132.665 PMC28471

[31] Hatakeyama M (2009) Helicobacter pylori and gastric carcinogenesis. J Gastroenterol 44: 239–248. 19271114 10.1007/s00535-009-0014-1

[32] Yamaoka Y (2010) Mechanisms of disease: Helicobacter pylori virulence factors. Nat Rev Gastroenterol Hepatol. England. pp. 629–641. 10.1038/nrgastro.2010.154 PMC3137895 20938460

[33] Furuta T, Shirai N, Takashima M, Xiao F, Hanai H, et al (2001) Effect of genotypic differences in CYP2C19 on cure rates for Helicobacter pylori infection by triple therapy with a proton pump inhibitor, amoxicillin, and clarithromycin. Clin Pharmacol Ther 69: 158–168. 11240980 10.1067/mcp.2001.113959

[34] Montalban C, Santon A, Boixeda D, Bellas C (2001) Regression of gastric high grade mucosa associated lymphoid tissue (MALT) lymphoma after Helicobacter pylori eradication. Gut 49: 584–587. 11559658 10.1136/gut.49.4.584 PMC1728454

[35] Michel M, Cooper N, Jean C, Frissora C, Bussel JB (2004) Does Helicobater pylori initiate or perpetuate immune thrombocytopenic purpura? Blood 103: 890–896. 12920031 10.1182/blood-2003-03-0900

[36] Rokkas T, Pistiolas D, Sechopoulos P, Robotis I, Margantinis G (2007) The long-term impact of Helicobacter pylori eradication on gastric histology: a systematic review and meta-analysis. Helicobacter 12 Suppl 232–38. 17991174 10.1111/j.1523-5378.2007.00563.x

[37] Nakamura Y (2007) The BioBank Japan Project. Clin Adv Hematol Oncol 5: 696–697. 17982410

[38] Kawaguchi T, Sho M, Tojo T, Yamato I, Nomi T, et al (2010) Clinical significance of prostate stem cell antigen expression in non-small cell lung cancer. Jpn J Clin Oncol 40: 319–326. 20085909 10.1093/jjco/hyp181

[39] Ono H, Hiraoka N, Lee YS, Woo SM, Lee WJ, et al (2012) Prostate stem cell antigen, a presumable organ-dependent tumor suppressor gene, is down-regulated in gallbladder carcinogenesis. Genes Chromosomes Cancer 51: 30–41. 21936014 10.1002/gcc.20928

[40] Reilly MP, Li M, He J, Ferguson JF, Stylianou IM, et al (2011) Identification of ADAMTS7 as a novel locus for coronary atherosclerosis and association of ABO with myocardial infarction in the presence of coronary atherosclerosis: two genome-wide association studies. Lancet 377: 383–392. 21239051 10.1016/S0140-6736(10)61996-4 PMC3297116

[41] Amundadottir L, Kraft P, Stolzenberg-Solomon RZ, Fuchs CS, Petersen GM, et al (2009) Genome-wide association study identifies variants in the ABO locus associated with susceptibility to pancreatic cancer. Nat Genet 41: 986–990. 19648918 10.1038/ng.429 PMC2839871

[42] Purcell S, Neale B, Todd-Brown K, Thomas L, Ferreira M, et al (2007) PLINK: a tool set for whole-genome association and population-based linkage analyses. Am J Hum Genet 81: 559–575. 17701901 10.1086/519795 PMC1950838

[43] Breslow N, Day N (1987) Statistical methods in cancer research. Volume II–The design and analysis of cohort studies. IARC Sci Publ: 1–406. 3329634

