# Correction: Impact of *PSCA* Variation on Gastric Ulcer Susceptibility

**DOI:** 10.1371/journal.pone.0115366

**Published:** 2014-12-05

**Authors:** The PLOS ONE Staff

There are errors in Table 2 of the published article. The correct version of Table 2 can be viewed as the Supporting Information file to this correction.

## Supporting Information

File S1 **Table 2.** Association of PSCA and ABO SNPs with gastric ulcer.
(XLSX)
