# Supplementary figures and images for: Impact of PSCA Variation on Gastric Ulcer Susceptibility
Source: PLoS One. 2013 May 21;8(5):e63698. doi: 10.1371/journal.pone.0063698 (PMC3660579; doi:10.1371/journal.pone.0063698)

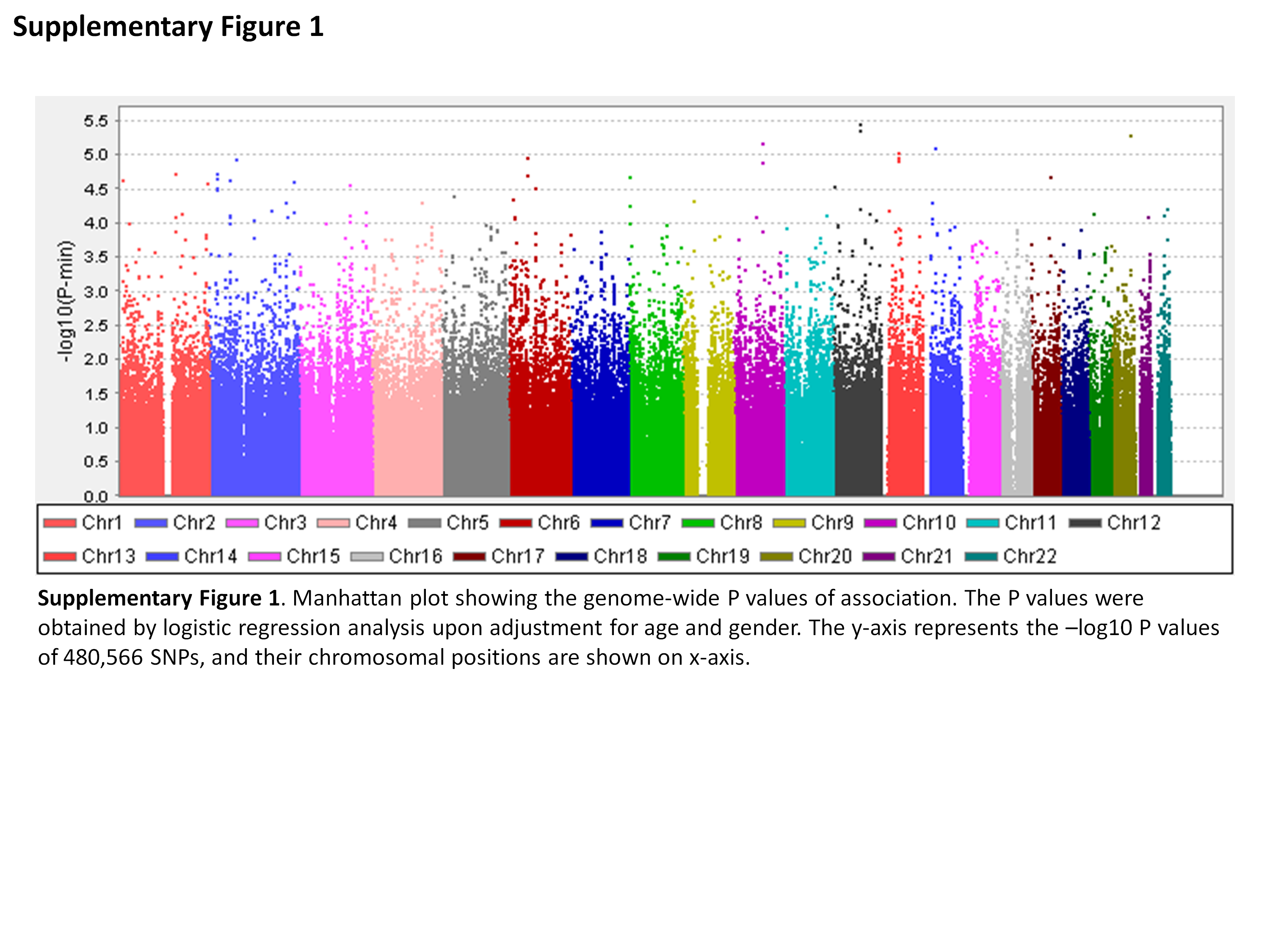

Supplement: Figure S1 — Manhattan plot showing the genome-wide P values of association. The P values were obtained by logistic regression analysis upon adjustment for age and gender. The y-axis represents the –log10 P values of 480,566 SNPs, and their chromosomal positions are shown on x-axis. (TIF) [file pone.0063698.s001.tif]
