# Supplementary material for: Impact of PSCA Variation on Gastric Ulcer Susceptibility
Source: PLoS One. 2013 May 21;8(5):e63698. doi: 10.1371/journal.pone.0063698 (PMC3660579; doi:10.1371/journal.pone.0063698)
Supplement: Supplementary file 2 [file pone.0063698.s002.docx]

Table S1. Genotype frequency of two SNPs in disease mix controls

Category	n	rs2294008							rs505922					
		CC	CT	TT	RAF	HWE[a]	P[b]	OR (95% C.I.)[c]	TT	CT	CC	RAF	HWE[a]	P[b]
colon cancer	1,302	174	633	495	0.377	0.20	0.792	0.99 (0.90-1.09)	393	649	260	0.551	0.79	0.681
breast cancer	1,423	224	629	569	0.379	0.02	0.927	1.00 (0.91-1.09)	424	694	305	0.542	0.50	0.704
diabetes	2,585	362	1164	1058	0.365	0.15	0.123	0.94 (0.87-1.02)	796	1273	516	0.554	0.86	0.410
arteriosclerosis obliterans	754	116	339	299	0.379	0.22	0.939	1.00 (0.89-1.12)	203	371	179	0.516	0.71	3.78 x 10[illegible]
atrial fibrillation	696	110	355	231	0.413	0.17	2.03×10^{-2}	1.15 (1.02-1.30)	227	323	146	0.558	0.12	0.427
brain infarction	1,309	156	590	563	0.345	0.94	1.92×10^{-3}	0.86 (0.78-0.95)	398	648	262	0.552	0.95	0.625
drug response	3,556	475	1645	1434	0.365	0.92	8.89×10^{-2}	0.94 (0.87-1.01)	1047	1760	748	0.542	0.87	0.643
amyotrophic lateral sclerosis	706	104	340	262	0.388	0.71	0.558	1.04 (0.92-1.17)	213	358	135	0.555	0.48	0.543
liver cancer	1,119	153	526	440	0.372	0.83	0.508	0.97 (0.87-1.07)	329	562	227	0.546	0.64	0.962
liver cirrhosis	1,217	156	575	486	0.364	0.49	0.188	0.94 (0.85-1.03)	346	586	283	0.526	0.25	9.73 x 10[illegible]
osteoporosis	1,368	188	611	569	0.361	0.24	9.19×10^{-2}	0.92 (0.84-1.01)	438	698	231	0.576	0.09	1.08 x 10[illegible]
fibroid	1,448	204	650	594	0.365	0.22	0.193	0.94 (0.86-1.03)	429	703	316	0.539	0.38	0.532
cervical cancer	155	17	63	75	0.313	0.50	1.69×10^{-2}	0.74 (0.58-0.95)	52	79	24	0.590	0.50	0.130
chronic hepatitis B	141	23	64	54	0.390	0.58	0.724	1.04 (0.82-1.34)	36	74	31	0.518	0.55	0.351
ovarian cancer	108	14	53	41	0.375	0.63	0.887	0.98 (0.74-1.30)	29	57	22	0.532	0.53	0.691
pulmonary tuberculosis	139	20	54	65	0.338	0.12	0.159	0.83 (0.65-1.08)	38	70	31	0.525	0.91	0.494
keloid	177	22	81	73	0.355	0.95	0.350	0.90 (0.72-1.13)	60	88	29	0.588	0.73	0.131
drug eruption	301	39	141	121	0.364	0.84	0.438	0.93 (0.78-1.11)	89	145	67	0.537	0.59	0.653
hematological cancer	81	7	36	37	0.313	0.67	7.98×10^{-2}	0.74 (0.53-1.04)	31	34	16	0.593	0.24	0.246
uterus cancer	134	11	65	58	0.325	0.22	6.51×10^{-2}	0.79 (0.60-1.02)	35	68	31	0.515	0.85	0.318
heat cramp	41	3	25	13	0.378	0.06	0.975	0.99 (0.63-1.56)	8	26	7	0.512	0.08	0.541
endometriosis	1,124	152	511	460	0.363	0.59	0.161	0.93 (0.84-1.03)	322	566	236	0.538	0.66	0.524
disease mix	19,884	2730	9150	7997	0.368	0.17	7.80×10^{-2}	0.95 (0.90-1.01)	5943	9832	4102	0.546	0.76	0.988
