# Supplementary material for: Impact of PSCA Variation on Gastric Ulcer Susceptibility
Source: PLoS One. 2013 May 21;8(5):e63698. doi: 10.1371/journal.pone.0063698 (PMC3660579; doi:10.1371/journal.pone.0063698)
Supplement: Supplementary file 3 [file pone.0063698.s003.docx]

rs6755780	2	54546994	7.65×10^{-5}	*SPTBN1*
rs5750033	22	33744017	7.79×10^{-5}	*ISX*
rs9289559	3	139573879	7.79×10^{-5}	*MRAS*
rs7945067	11	119183655	7.94×10^{-5}	*LOC390255*
rs6740698	2	54577245	7.96×10^{-5}	*SPTBN1*
rs7279123	21	35336957	7.97×10^{-5}	*RUNX1*
rs10509101	10	60804341	8.03×10^{-5}	*FAM13C1*
rs1026882	2	213095634	8.13×10^{-5}	*ERBB4*
rs12049103	1	156288841	8.18×10^{-5}	*KIRREL*
rs1495982	6	19498168	8.20×10^{-5}	*LOC100131913*
rs17212261	14	25434780	8.41×10^{-5}	*LOC401767*
rs9460351	6	19509397	8.63×10^{-5}	*LOC100131913*
rs34520190	2	120665579	8.92×10^{-5}	*LOC729669*
rs7303593	12	118200036	9.07×10^{-5}	*LOC144742*
rs9851969	3	138495078	9.85×10^{-5}	*IL20RB*
rs11247946	1	26591702	9.89×10^{-5}	*LIN28*
rs6806589	3	76231050	9.91×10^{-5}	*LOC100131827*

We analyzed 1,862 gastric ulcer cases and 17,482 controls in the analysis. Chr.,chromosome; Postion in the NCBI Build 36.3. [a] P values and ORs were adjusted for age and gender by logistic regression analysis.
